# Glutamine Metabolism and Metabolic Profiling Using 7 T CRT‐FID MRSI in Focal Epilepsy

**DOI:** 10.1111/ene.70343

**Published:** 2025-09-12

**Authors:** Stefanie Chambers, Philipp Lazen, Matej Hotka, Haniye Shayeste, Matthias Tomschik, Jonathan Wais, Vitalij Zeiser, Lukas Hingerl, Bernhard Strasser, Lukas Haider, Tatjana Traub‐Weidinger, Christoph Baumgartner, Johannes Koren, Florian Mayer, Martha Feucht, Christian Dorfer, Ekaterina Pataraia, Wolfgang Bogner, Siegfried Trattnig, Gregor Kasprian, Karl Rössler, Gilbert Hangel

**Affiliations:** ^1^ Department of Neurosurgery Medical University of Vienna Austria; ^2^ MRCE, Department of Biomedical Imaging and Image‐Guided Therapy Medical University of Vienna Austria; ^3^ Christian Doppler Laboratory for MR Imaging Biomarkers Vienna Austria; ^4^ Center of Physiology and Pharmacology, Department of Neurophysiology and Neuropharmacology Medical University of Vienna Austria; ^5^ Division of Physiology, Department of Pharmacology, Physiology, and Microbiology Karl Landsteiner University of Health Sciences, Krems Austria; ^6^ Division of Neuroradiology and Musculoskeletal Radiology, Department of Biomedical Imaging and Image‐Guided Therapy Medical University of Vienna Austria; ^7^ NMR Research Unit, Queen Square Multiple Sclerosis Centre, Queen Square Institute of Neurology University College London UK; ^8^ Comprehensive Center for Clinical Neurosciences and Mental Health Medical University of Vienna Vienna Austria; ^9^ Division of Nuclear Medicine, Department of Biomedical Imaging and Image‐Guided Therapy Medical University of Vienna Austria; ^10^ Department of Neurology, Klinik Hietzing Vienna Austria; ^11^ Department of Pediatrics and Adolescent Medicine, Center for Rare and Complex Epilepsies, Member of ERN EpiCARE Medical University of Vienna Austria; ^12^ Department of Neurology Medical University of Vienna Austria; ^13^ Functional Imaging Laboratory, UCL Queen Square, Institute of Neurology University College London UK

**Keywords:** 7 T, epilepsy, glutamine, spectroscopy, ultra‐high‐field MRSI

## Abstract

**Background:**

Approximately one‐third of people with epilepsy (PWE) remain drug‐resistant. In these cases, surgical resection of the epileptogenic zone may significantly reduce or eliminate seizures. Surgery necessitates precise delineation of the epileptogenic zone (EZ) which proves especially challenging in the 20% of PWE that remain MRI‐negative. The purpose of this study was to analyze the feasibility and robustness of ultra‐high‐field MRSI in identifying and characterizing pathologies in focal epilepsy. In addition, the relationship of glutamate and glutamine was evaluated in the EZ.

**Methods:**

Fifty‐six people with focal epilepsy were prospectively measured using 7 T concentric ring trajectory direct acquisition of free‐induction‐decay MRSI, which generated whole‐brain metabolic maps with an isotropic resolution of 3.4mm^3^. After exclusion criteria were applied, we assessed metabolite ratios in 15 lesional and 14 MRI‐negative PWE.

**Results:**

In the lesional group, metabolic alterations in the suspected EZ were present in 86.7% of maps normalized to N‐acetyl‐aspartate, whereas this was reduced to 80% in creatine ratios. Metabolites with the highest consistency in the lesional group included myo‐inositol and choline, showing increases in 92.3% of PWE. In MRI‐negative patients, changes were heterogeneous, with a detection rate of 57.1%. We also observed a tendency toward an inverse relationship of glutamate to glutamine in the EZ, with increases of glutamine in PWE with lower seizure frequencies, contrasting glutamate increases in higher seizure frequencies.

**Conclusion:**

Our preliminary analysis suggests that 7 T CRT‐FID MRSI shows promise not only in identifying metabolic alterations in focal epilepsy but may also provide insights into disease pathomechanisms.

## Graphical abstract

Using 7 T Concentric Ring Trajectory‐Free Induction Decay (CRT‐FID) Magnetic Resonance Spectroscopy Imaging (MRSI), generating full‐brain metabolic maps with a 3.4 mm isotropic resolution in people with epilepsy is possible for the first time. In this study, we could demonstrate that 7 T MRSI shows promise as a feasible method for detecting metabolic alterations in 15 lesional and 14 MRI‐negative patients, yielding 86.67% detection rates when normalized to N‐acetyl‐aspartate, with the highest rates seen in myo‐inositol and choline ratios in lesional epilepsy at 92.3% and lower detection rates of 57.1% in MRI‐negative PWE. Furthermore, our preliminary results indicate that glutamate and glutamine share an inverse relationship in the epileptogenic zone, potentially indicating altered energy metabolism in epilepsy.

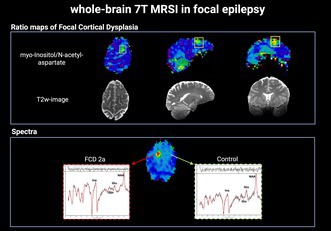

**Abbreviations**
CRLB: Cramér‐Rao lower bound
CRT: concentric ring trajectories
EEG: electroencephalography
EZ: epileptogenic zone
FCD: focal cortical dysplasia
FID: free induction decay
FLAIR: fluid‐attenuated inversion recovery
FOV: field of view
FWHM: full width at half maximum
Gln: glutamine
Glu: glutamate
HS: hippocampal sclerosis
LEAT: low‐grade epilepsy‐associated neuroepithelial tumor
M2RAGE: magnetization‐prepared 2 rapid acquisition gradient echoes
MCD: malformations of cortical development
mIns: myo‐inositol
MRI: magnetic resonance imaging
MRSI: magnetic resonance spectroscopic imaging
PET: positron emission tomography
PW(F)E: people with (focal) epilepsy
ROI: region of interest
SAR: specific absorption rate
SNR: signal‐to‐noise ratio
tCho: choline‐containing compounds
tCr: total creatine, creatine + phosphocreatine
tNAA: N‐acetyl‐aspartate + *N*‐acetyl‐aspartyl glutamate
UHF: ultra‐high‐field
WET: water suppression enhanced through T_1_ effects
WMS: fluid and white‐matter suppressed

## 1 Introduction

Epilepsy is caused by a wide array of underlying pathologies, and approximately one‐third of people with epilepsy (PWE) remain drug‐resistant [1] despite multiple antiseizure medications (ASM). In people with focal epilepsy (PWFE), surgery may prove beneficial, but postoperative seizure freedom heavily depends on total resection of the epileptogenic zone (EZ) [2]. In some cases, this may extend past visible lesion borders or remain undetectable in structural MRI (Magnetic Resonance Imaging), that is, MRI‐negative focal epilepsy. Though a recent implementation of the 7 T MRI consensus protocol [3] for epilepsy showed that the application of ultra‐high‐field strengths yields a diagnostic gain of up to 50% in focal epilepsy compared to dedicated 3 T epilepsy protocols [4], the remaining medically nonresponsive patients without macroscopically detectable lesions pose a challenge in surgical planning. In such cases, imaging modalities shedding light on brain functionality by measuring metabolites [5] (such as positron‐emission tomography (PET) or magnetic resonance spectroscopy (MRS)) may aid in identifying subtle pathologies that are otherwise blind to structural MRI. However, given that placement of the ROI is necessary due to the circumscribed coverage of MRS, its practicality in MRI‐negative cases in which the epileptogenic zone (EZ) is frequently unclear is limited. As these patients make up 20% of PWE [6], there is a pressing need for the application of whole‐brain metabolic maps with larger volume coverage while maintaining adequate spectral resolution.

To this end, we developed a fast, high‐resolution MRSI method based on concentric ring trajectory direct acquisition of free‐induction‐decay (CRT‐FID) [7, 8, 9, 10], enabling the generation of 3D whole‐brain maps of 14 neurochemicals at 3.4 mm isotropic resolution within 15 min, making high‐resolution whole‐brain MRSI feasible in PWFE for the first time. Thus far, we have successfully applied this method to neurological diseases such as multiple sclerosis [11, 12] and brain tumors [13, 14].

MRS in epilepsy has frequently been limited by overlapping resonance frequencies of metabolites due to low spatial resolution and signal‐to‐noise ratios (SNR) at standard clinical field strengths (i.e., 1.5 T and 3 T) [15, 16] and by the use of single‐voxel and multislice spectroscopy [15, 16] which is useful in patients with visible MRI lesions and in whom ROI placement is evident. MRI‐negative cases, however, require whole‐brain coverage and, although this has been achieved at lower field strengths (3 T) [17], the decreased spatial resolution at 3 T limits its application. Groups investigating metabolic alterations using MRS in malformations of cortical development (MCD) [16, 18] have found N‐acetyl‐aspartate (NAA), synthesized in neuronal mitochondria and a measure of healthy neuronal function [19, 20] to be significantly decreased in focal cortical dysplasia (FCD) and epileptic lesions [16, 17, 19, 20] which has been shown to correlate with disease duration [21]. Decreases in NAA are often mirrored by increases in choline (Cho) [18], a marker for cell membrane turnover, frequently altered in tumors, and myo‐inositol (mIns) [16], reflecting astroglial hypertrophy. However, the metabolic landscape throughout the EZ remains ambiguous, highlighting the complexity of dynamic processes inherent to epileptic metabolic activity as well as its numerous drivers. Some metabolites have been shown to be dependent on clinical parameters, such as the seizure frequency, as seen in creatine [16], an energy buffer, for which increases [16, 22] or decreases [18] have been described in the EZ. Another key metabolite and neurotransmitter inherently dependent on clinical variables is glutamate (Glu), closely linked to glutamine (Gln) through the glutamate‐glutamine cycle. Increases [23], unchanged concentrations [24], and decreases for Glx (glutamate + glutamine) have been described interictally, though previous in vivo research has frequently been limited by the overlapping resonance frequencies of these neurochemicals, compounding them as “Glx.” However, these metabolites may act countervailingly in energy metabolism [23, 25], necessitating their spectral separation for adequate interpretation. This is supported by the findings of the application of 7 T CRT‐FID MRSI in tumors, showing decreases of peritumoral Glu/Gln ratios in tumor‐associated epilepsy (TAE), contrasting without TAE [26].

The heterogeneity of the changes described above highlights the need for ultra‐high‐resolution, whole‐brain MRSI. The aim of our study is to qualitatively analyze the feasibility of 7 T CRT‐FID MRSI in focal epilepsy to generate high‐resolution 3D maps spanning the cerebrum. Specifically, we investigated its utility in the identification and characterization of metabolic alterations over pathologies and assessed glutamate and glutamine in the EZ.

## 2 Methods

### 2.1 Patient Selection Criteria and Clinical Work‐Up

**FIGURE 1:**
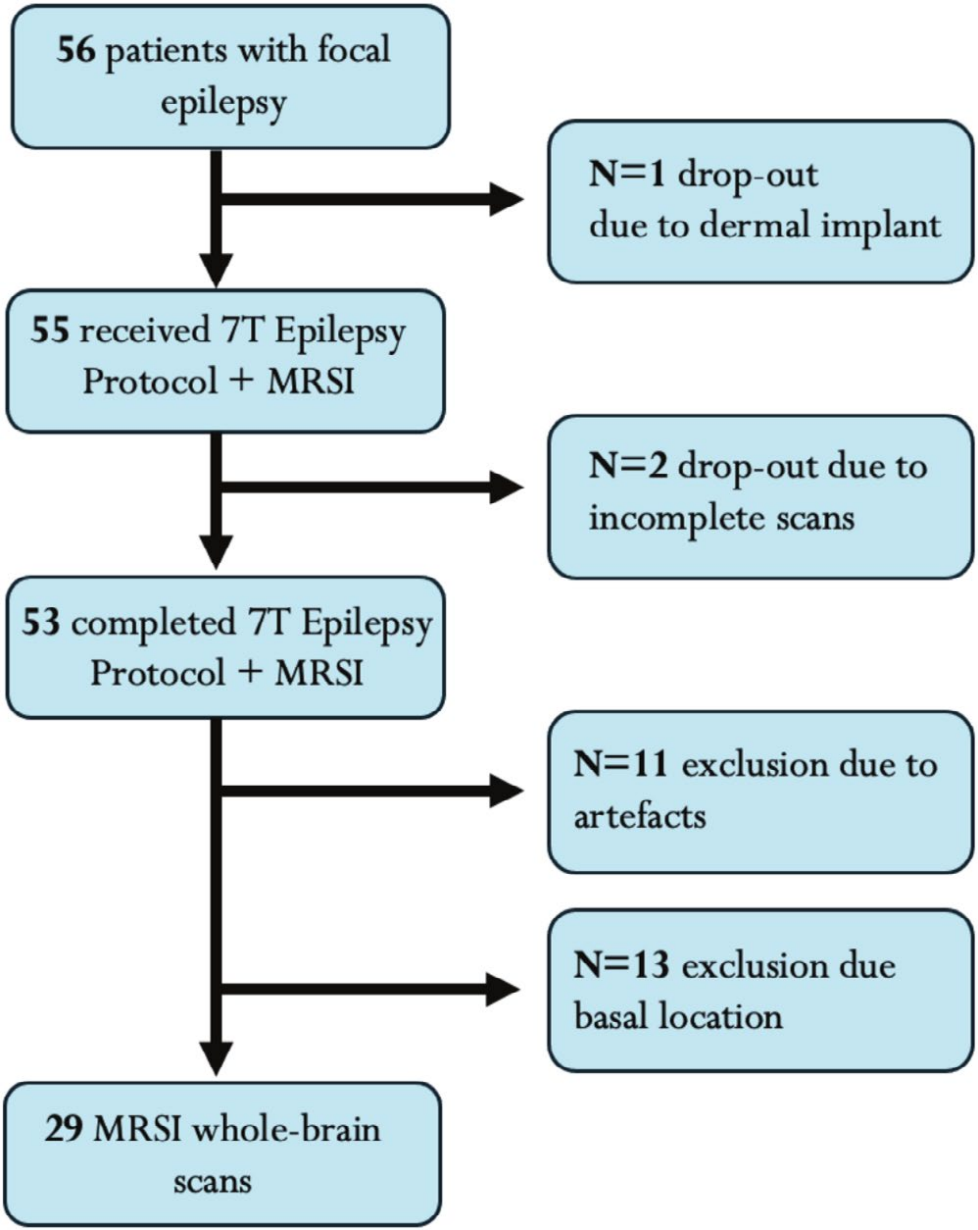
Flowchart of recruitment and drop‐outs during measurement and analysis. Exclusion criteria included the presence of movement or lipid artifacts in derived maps and basally located (temporomesial) EZ, due to impeding field inhomogeneities. 7 T, 7 Tesla; EZ, epileptogenic zone; MRSI, Magnetic Resonance Spectroscopy Imaging.

Following the approval of the institutional board of review (EK 1039/2020) 56 PWFE of at least 12 years of age (14–57 years, 26 females) were enrolled in this prospective study following written, informed consent (or that of their guardian). Exclusion criteria included claustrophobia, metal implants, pregnancies, and vascular etiologies. PWFE who were included in this study had all undergone extensive presurgical clinical work‐up [27, 28] at the Medical University of Vienna or Department of Neurology, Klinik Hietzing, Vienna, including a prolonged video‐electroencephalogram (VEEG), assessment of seizure semiology, neuropsychological testing, a 3 T MRI using a dedicated epilepsy protocol and [^18^F]Fluorodeoxyglucose (FDG)‐PET to localize the suspected EZ. For the purpose of this preliminary analysis, the suspected EZ was extended to the whole affected lobe. All patients had been presented at interdisciplinary board meetings for the evaluation of surgical candidacy. Fifty‐six patients were prospectively measured from July 2020 to July 2024 prior to surgery, and of these, ten were consequently operated on, providing histopathological diagnoses. We dichotomized patients into lesional (either suspected in neuroradiological assessment or histopathologically confirmed) and nonlesional (i.e., MRI‐negative) groups. The clinical parameters included the seizure frequency, quantified using the seizure frequency score [29] (categorized into daily = 9–10, weekly = 8, monthly = 7 and yearly occurring seizures = 5–6) and epilepsy duration (Table 1). A detailed overview of the clinical characteristics of included patients is provided in Suppl. Table S1. A flowchart of patient recruitment is provided in Figure 1.

**TABLE 1:** Cohort summary.

Overall (*N* = 29)	
**Age (years)**	
Mean (SD)	26.3 (9.7)
Range	14–57
**Sex**	
Female	13 (44.83%)
Male	16 (55.17%)
**Etiology**	
MRI Negative	14 (48.28%)
Malformation of Cortical Development	15 (51.72%)
**Histopathological verification**	10/15 (66.67%)
**Suspected EZ**	
Temporal	8 (27.59%)
Extra‐Temporal	21 (72.41%)
**Seizure Frequency**	
Median (Range)	7.5 (2–10)
**Duration of Epilepsy (years)**	
Mean (SD)	14 (6.97)
Range	2–27

*Note:* Overview of patient cohort. MRI‐negative excluded incidental findings unrelated to the EZ. An extensive table of included pathologies of malformations of cortical development and affected lobes in extra‐temporal epilepsy is provided in (Table S1).

### 2.2 Measurement Protocol

The 3D‐MRSI protocol was acquired with a 7 T scanner (Siemens Healthineers, Magnetom Plus, Erlangen, Germany) with a 32Rx/1Tx‐coil (Nova Medical, Wilmington, MA, USA). The MRSI sequence [9] using 2D‐CRT featured a 64 × 64 × 39 measurement matrix and a 220 × 220 × 133 mm^3^ field of view (FOV) resulting in 3.4 mm isotropic resolution acquired in 15 min. It used FID acquisition with a 39° flip angle and an acquisition delay of 1.3 ms. A TR of 450 ms and WET water suppression [10] allowed for an SNR‐optimized readout of 345 ms and a 2778 Hz spectral bandwidth. Details according to Minimum Reporting Standards in in vivo Magnetic Spectroscopy (MRSinMRS) [30] are supplied in Table S4. In addition to the MRSI protocol, morphological sequences included MP2RAGE (Magnetization Prepared 2 Rapid Acquisition Gradient Echoes), coronal hippocampal T2‐weighted images, 3D FLAIR (Fluid‐Attenuated Inversion Recovery), 3D WMS (White Matter Suppressed), and transversal SWI (Susceptibility‐Weighted Imaging), as previously published [4].

### 2.3 Data Post‐Processing

Following the acquisition, MRSI data were processed with an in‐house pipeline [31] including lipid signal removal through L2‐regularization [32] using Matlab (R2013a, MathWorks, MA, USA), Bash (v4.2.25, Free Software Foundation, Boston, MA, USA) and MINC Toolkit (MINC tools, v2.0, McGonnell Brain Imaging Center, Montreal, QC, Canada). Resulting voxel spectra were quantified using LCModel (v6.3–1, LCMODEL Inc., ONT, CA) with a basis set of 14 components and a macromolecular baseline [33] in a spectral range of 1.8–4.2 ppm [13]. Due to their relevance in epilepsy, tNAA, tCr, tCho, mIns, Glu, and Gln were used in our analysis.

### 2.4 Data Evaluation

Voxel‐wise spectral quality was automatically assessed using the pseudo‐replica method for SNR and full‐width‐at‐half‐maximum (FWHM) of tCr at 3.02 ppm and the fitting quality using the Cramér‐Rao lower bounds (CRLBs) in all metabolites. Voxels were excluded if the tCr SNR is less than 5 or the tCr FWHM is greater than 0.15 ppm [13].

In addition to the automatized filtering of spectra, the overall quality of the metabolic maps was visually reviewed by a trained reader (S.C.) and referred to an MRI physicist (G.H.) in the presence of lipid or movement artifacts. Metabolite maps that did not fulfill the quality criteria were excluded from further analysis. Equally, maps in which the EZ was located temporo‐mesially were not included due to the interference of basal field inhomogeneities on our 7 T scanner (Figure 1).

The remaining metabolite maps were analyzed as ratio maps normalized to tNAA because of three main advantages: due to frequently described decreases of NAA in epilepsy, normalizing to this metabolite increases the detectability of potential hotspots; and second, as NAA is exclusively synthesized in neurons, normalization to NAA allows for a proxy for neuronal density even in pathological tissue with increased gliosis or atrophy. Last, in contrast to creatine, this metabolite has, to date, not been shown to be sensitive to clinical parameters such as seizure frequency. However, normalization to creatine does have the advantage of being relatively stable throughout the brain with a robust signal, for which reason a comparison of both ratios will be provided in this work.

To assess the sensitivity of MRSI in identifying altered metabolic status, metabolite maps were visually assessed in the region of interest (ROI), representing the suspected EZ, as defined in the clinical work‐up compared to the contralateral hemisphere. Changes were categorically defined as the following:

- “increase” = hotspot of metabolite in amplitude ratio map in ROI compared to contralateral corresponding localization
- “decrease” = coldspot of metabolite in amplitude ratio map in ROI compared to contralateral corresponding localization
- “MRSI‐negative” = no visible changes over the entirety of the assessed metabolite panel
- “stable” = no change in the EZ of a specific metabolite, despite alterations of other metabolites in the EZ

In a second step, the metabolic patterns were qualitatively assessed in relation to the underlying pathologies and related to seizure frequency. Unidirectionality of alterations (increase or decrease) was considered stable. Due to the role of Glu and Gln in energy metabolism, these were specifically assessed in the EZ.

### 2.5 Statistical Analysis

Due to the explorative and qualitative scope of this study, results are largely limited to descriptive statistics. Given the low sample size and heterogeneity of the data, a Fisher's Exact Test was applied in the evaluation of glutamate and glutamine ratios in the EZ. To partially account for the absence of covariate adjustment, the mean and median values of age and drug load are reported. A paired t‐test was conducted to assess differences between the groups. Statistical significance of the observed results is considered for *p* < 0.05.

## 3 Results

### 3.1 Detection of Metabolic Alterations in the EZ

**FIGURE 2:**
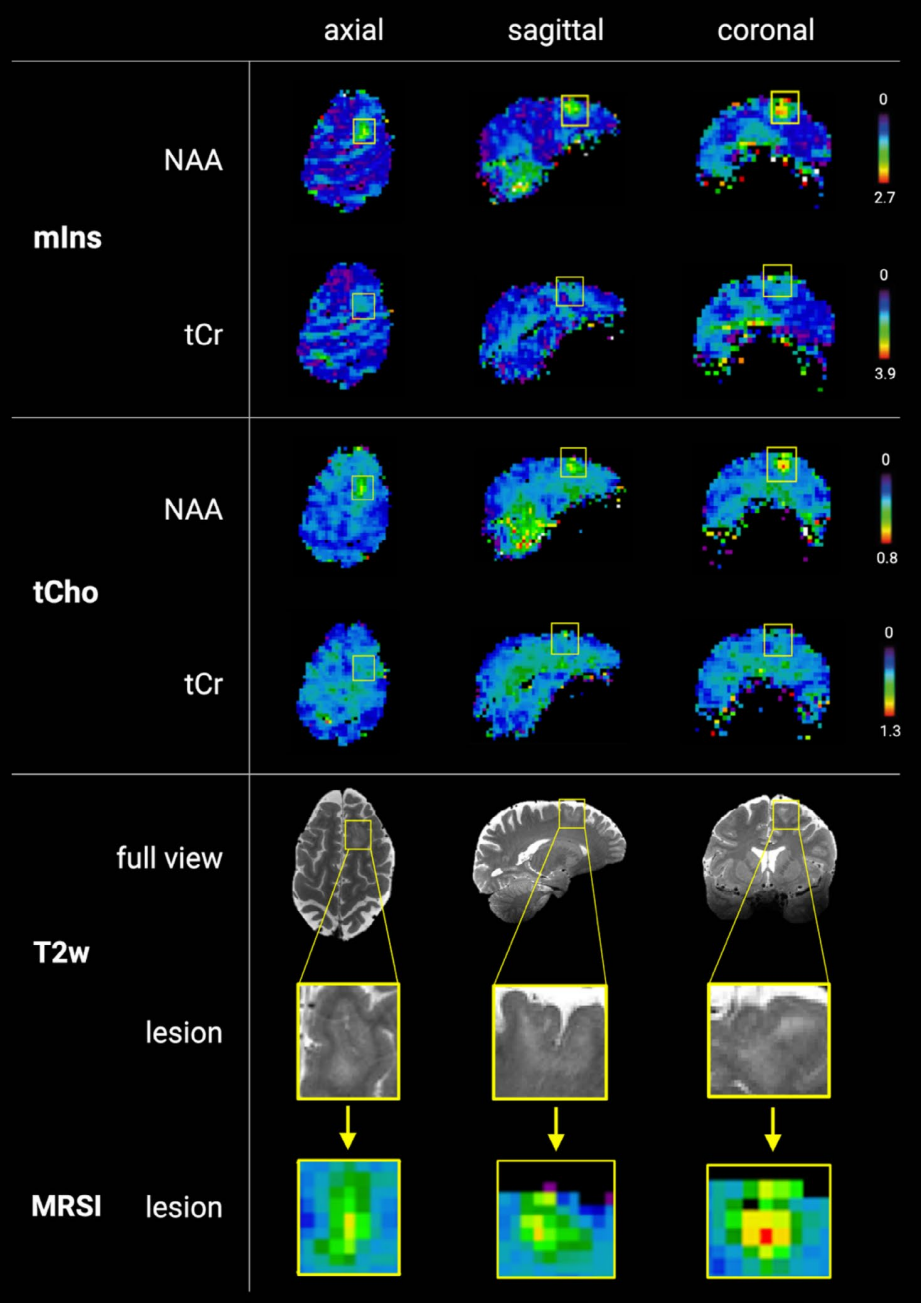
Ratio maps of mIns and tCho normalized to tCr and NAA showcasing the detectability of metabolic alterations in FCD. Metabolic ratio maps of patient 2 with FCD Type 2b. A mIns to tCr and tNAA and B tCho to tCr and tNAA. The corresponding lesions in 7 T T2‐weighted images are depicted in C. While the ratios normalized to NAA clearly show a metabolic hotspot corresponding to the lesion location, as highlighted in the yellow box, the structural abnormality remains nearly undetected in mIns/tCr and inconspicuous in tCho/tCr. 7 T, 7 Tesla; Cr, creatine; MIns, Myo‐inositol; NAA, *N*‐acetyl‐aspartate + *N*‐acetyl‐aspartyl glutamate; T2w, T2‐weighted; tCho, total choline.

**FIGURE 3:**
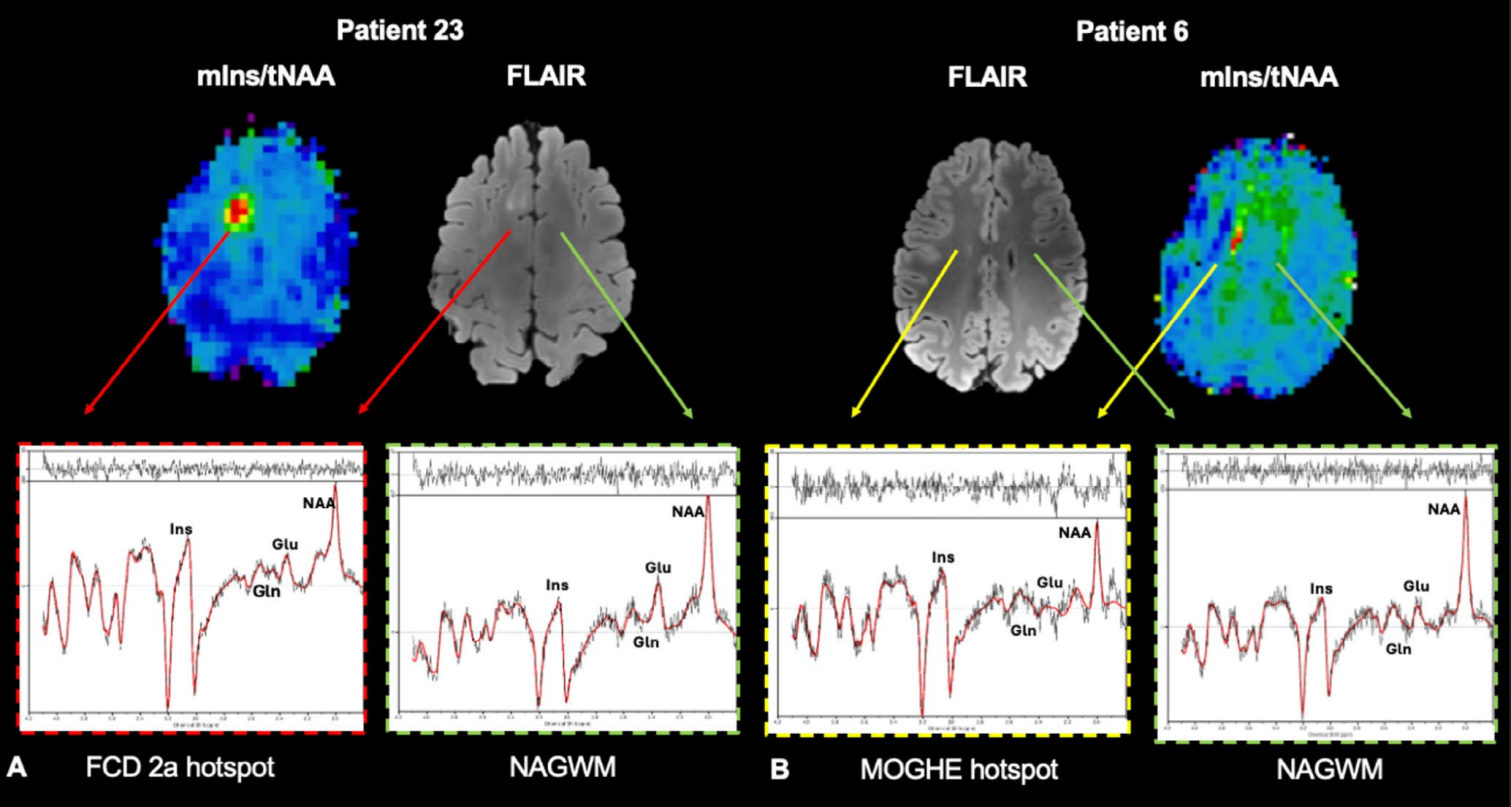
Exemplary spectra in EZ and contralateral ROI. (A) Histopathologically verified FCD and normal‐appearing gray‐matter contralaterally. (B) Histopathologically verified MOGHE in the left frontal lobe and contralateral NAGWM. The red arrows point to the corresponding spectra of the FCD lesion, the green arrow to spectra in normal‐appearing gray matter, and the yellow arrows to exemplary spectra in a MOGHE hotspot. T2‐weighted FLAIR images of the lesions are provided in the center. FCD, Focal Cortical Dysplasia; MOGHE, Mild Malformation of Cortical Development with Oligodendroglial Hyperplasia and Epilepsy; NAGWM, Normal‐appearing gray and white matter.

In the lesional group, metabolic alterations in suspected EZ were found in 13/15 (86.67%) of the maps in ratios normalized to NAA; this was reduced to 12/15 (80%) when normalized to creatine (see Figure 2). The assessed pathologies included eight cases of FCD, two mild malformations of cortical development (mMCD), two cases of polymicrogyria, one mild malformation of cortical development with oligodendroglial hyperplasia and epilepsy (MOGHE), a patient with blurred GM/WM boundaries and chronic epilepsy associated changes, and a patient with distinct focal atrophy of the temporolateral lobe with suspected MCD. A summary of metabolite alterations is provided in Table 2. Histopathological verification of the above‐mentioned pathologies was available in 10/15 (66.67%) (Table 1). Unidentified lesions using MRSI included histopathologically confirmed MCD and suspected polymicrogyria. As illustrated in the exemplary spectra of patients with FCD and MOGHE in Figure 3, metabolic patterns within the suspected EZ visibly differed from normal‐appearing gray and white matter (NAGWM).

**TABLE 2:** Summary of qualitative assessment of metabolic alterations in EZ.

Patient	Diagnosis	Glu/tNAA	Gln/tNAA	Ins/tNAA	tCho/tNAA	tCr/tNAA	Glu/tCr	Gln/tCr	Ins/tCr	Cho/tCr	tNAA/tCr
**1**	FCD 1b	_	−	+	+	+	_	_	+	+	−
**2**	FCD 2b	−	+	+	+	+	−	_	_	_	−
**3**	FCD 2a	_	+	+	+	+	−	+	+	+	_
**4**	MRI‐negative	+	_	+	+	+	+	−	+	+	−
**5**	SCN1a Mutation	+	+	−	−	_	+	+	_	_	+
**6**	MOGHE	_	m.v.	+	+	_	/	m.v.	/	/	/
**7**	MCD	/	/	/	/	/	/	/	/	/	/
**8**	chronic epileptic changes	+	+	+	+	_	_	_	_	+	−
**9**	Polymicrogyria	+	+	+	+	+	+	+	+	+	−
**10**	MRI‐negative	/	/	/	/	/	/	/	/	/	/
**11**	MRI‐negative	+	_	_	_	−	/	/	/	/	/
**12**	mMCD Type II	_	+	+	+	+	−	_	_	_	−
**13**	MRI‐negative	+	_	−	−	−	+	+	_	_	_
**14**	MRI‐negative	/	/	/	/	/	/	/	/	/	/
**15**	MRI‐negative	/	/	/	/	/	/	/	/	/	/
**16**	suspected Polymicrogyria	/	/	/	/	/	/	/	/	/	/
**17**	FCD 2a	+	_	_	_	_	_	_	_	_	_
**18**	FCD 2a	+	−	+	+	_	+	+	+	+	_
**19**	MRI‐negative	/	/	/	/	/	/	/	/	/	/
**20**	MRI‐negative	/	/	/	/	/	/	/	/	/	/
**21**	MRI‐negative	_	_	_	_	_	+	_	+	+	_
**22**	MRI‐negative	−	_	−	−	_	−	−	−	−	_
**23**	suspected FCD	−	+	+	+	+	−	_	+	_	−
**24**	MRI‐negative	+	_	+	+	+	+	_	+	_	−
**25**	MRI‐negative	/	/	/	/	/	/	/	/	/	/
**26**	suspected FCD	_	+	+	+	+	−	_	+	+	−
**27**	suspected FCD	−	+	+	+	_	−	+	_	_	_
**28**	MRI: focal temporal atrophy	+	+	+	+	+	_	_	_	−	−
**29**	MRI‐negative	+	+	+	+	−	+	+	+	+	+

*Note:* + = increase in EZ, − = decrease in EZ, _ = stable value of metabolites in EZ, / = MRSI‐negative (no visual changes in all assessed metabolites in EZ), m.v. = missing value. Diagnosis refers to either histopathological assessment or MRI diagnosis (i.e., suspected).

Abbreviations: Cr, creatine; EZ, epileptogenic zone; Gln, glutamine; Glu, glutamate; mIns, Myo‐inositol; NAA, *N*‐acetyl‐aspartate + *N*‐acetyl‐aspartyl glutamate; tCho, total choline.

Of the 14 PWE who were MRI‐negative, 42.9% (6/14) remained MRSI‐negative, highlighting that metabolic alterations were present in the majority of MRI‐negative patients, though in markedly fewer than in lesional patients (Table S3).

### 3.2 Metabolic Alterations Across Pathologies

**FIGURE 4:**
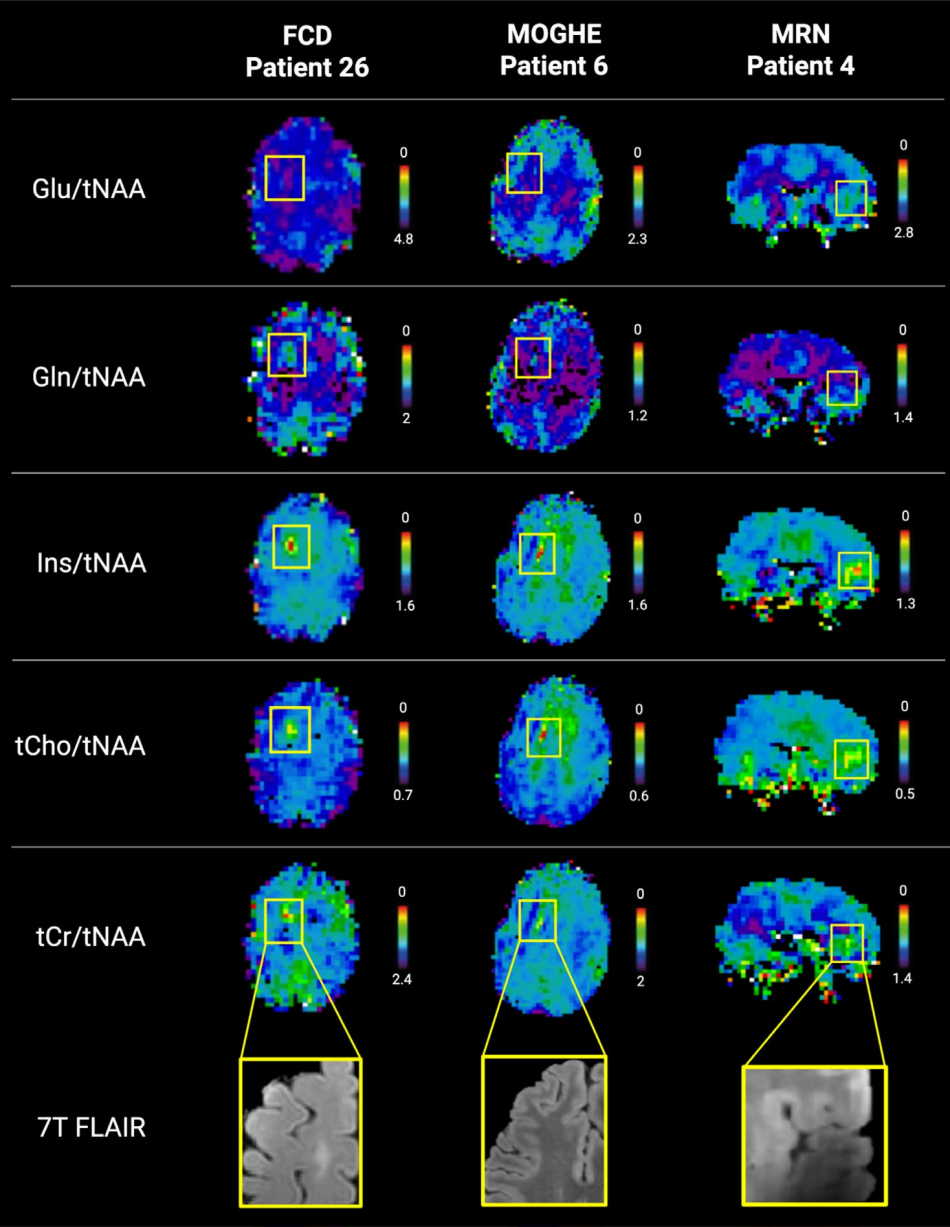
Overview of metabolic alterations in focal epilepsy. Metabolic alterations are highlighted by the yellow box, and the corresponding structural T2‐weighted FLAIR images are provided in the last row. First row: Axial metabolic ratio maps of patient 26 of Glu, Gln, Ins, tCho, and tCr normalized to NAA with histopathologically verified FCD 2a. Second row: Axial metabolite ratio maps in patient 6 with MOGHE in the left frontal lobe. Third row: Coronal metabolite ratio maps in patient 4 with MRI‐negative focal epilepsy. Based on the extensive clinical work‐up, the suspected EZ was located in the right insula, in which MRSI showed metabolite ratios of mIns and tCho, albeit less circumscribed than in the lesionally confirmed patients. Field inhomogeneities visible in 7 T FLAIR. 7 T, 7 Tesla; Cr, creatine; EZ, epileptogenic zone; FCD, Focal Cortical Dysplasia; MIns, Myo‐inositol; MOGHE, Mild malformation of cortical development with oligodendroglial hyperplasia and epilepsy; MRN, MRI‐negative; NAA, *N*‐acetyl‐aspartate + *N*‐acetyl‐aspartyl glutamate; NAGWM, Normal appearing gray‐and‐white matter; T2w, T2‐weighted; tCho, total choline.

In a more detailed analysis of metabolic changes, we identified alterations over numerous pathologies (Figure 4).

**FIGURE 5:**
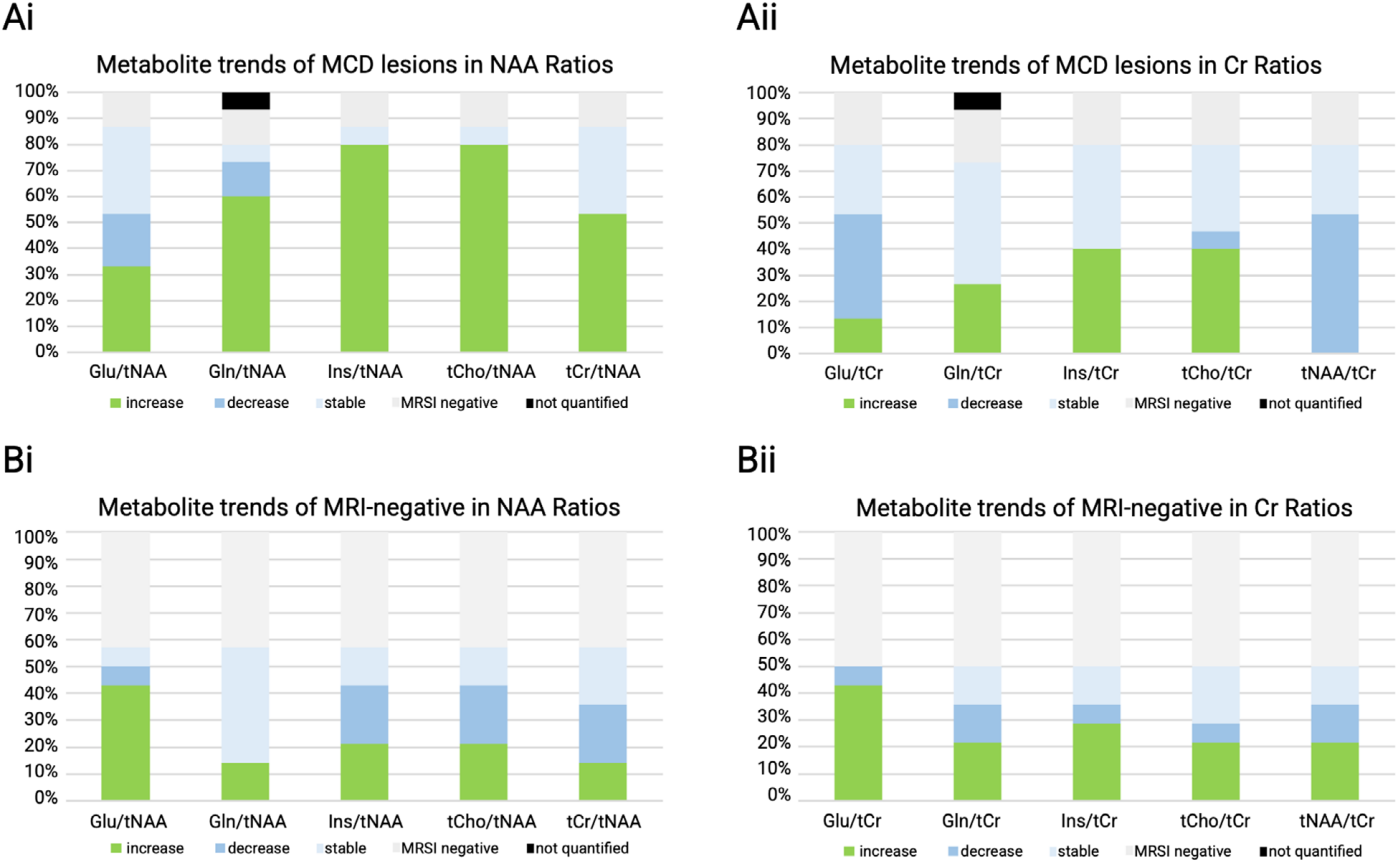
Metabolic alterations in MCD lesions and MRI‐negative cases. Ai and Aii illustrate the metabolic alterations of Glu, Gln, mIns, tCho, and tCr normalized to NAA and tCr, respectively, showcasing the increased detection rate of metabolic alterations in ratios normalized to NAA. Specifically, normalizing to mIns and tCho shows high sensitivity and stability in uncovering and characterizing MCD lesions. Bi and Bii in MRI‐negative patients. In normalization to either metabolite, 60% showed alterations, albeit with heterogeneous patterns. Overall, normalizing to creatine uncovered markedly fewer metabolic alterations compared to NAA. “Stable ”refers to no apparent change in the EZ of a specific metabolite, despite alterations of other metabolites in the ROI, whereas “MRSI‐negative ”refers to none of the investigated metabolites showing altered concentrations. Gln, glutamine; Glu, glutamate; Ins, Myo‐inositol; MCD, malformations of cortical development; ROI, region of interest; tCho, choline; tCr, creatine; tNAA, *N*‐acetyl‐aspartate and N‐acetyl‐aspartyl glutamate.

Due to the higher detectability of alterations, further analysis was conducted only in maps normalized to NAA. In patients with visible lesions, circumscribed increases of tCho and mIns were observed in 12/13 (92.3%) (excluding “MRSI negative” patients, that is, in which none of investigated metabolite ratios showed alterations), while no decreases were detected, as illustrated in Figure 5. Other fitted metabolites, such as Glu and Gln, showed varying directionality of changes, with Glu increases in 5/13 (38.5%), decreases in 3/13 (23.1%), and no changes in 5/13 (38.5%) of the investigated maps. Gln showed higher rates of increases in 9/13 (69.2%) and lower rates of decreases in 2/13 (15.4%). No visible alterations for Gln were seen in 1/13 (7.7%). Ratio maps of tCr/tNAA showed an increase in 8/13 (61.5%), whereas in 5/13 (38.5%), no changes were visually discernible.

The metabolic pattern in MRI‐negative patients was markedly more heterogeneous, with an overall detection rate of 8/14 (57.1%). Furthermore, all metabolites showed multidirectionality of changes (Figure 5, B i and ii), contrasting the findings above. As histopathology and postoperative seizure freedom of the suspected EZ was available in only one patient, the interpretation of these results remains questionable.

Overall, metabolic maps show high heterogeneity of metabolic alterations throughout patients, summarized in Table S2,S3. Furthermore, the directionality of metabolite changes seems to be, at least partially, dependent on clinical parameters.

### 3.3 Glutamate and Glutamine in the EZ

**FIGURE 6:**
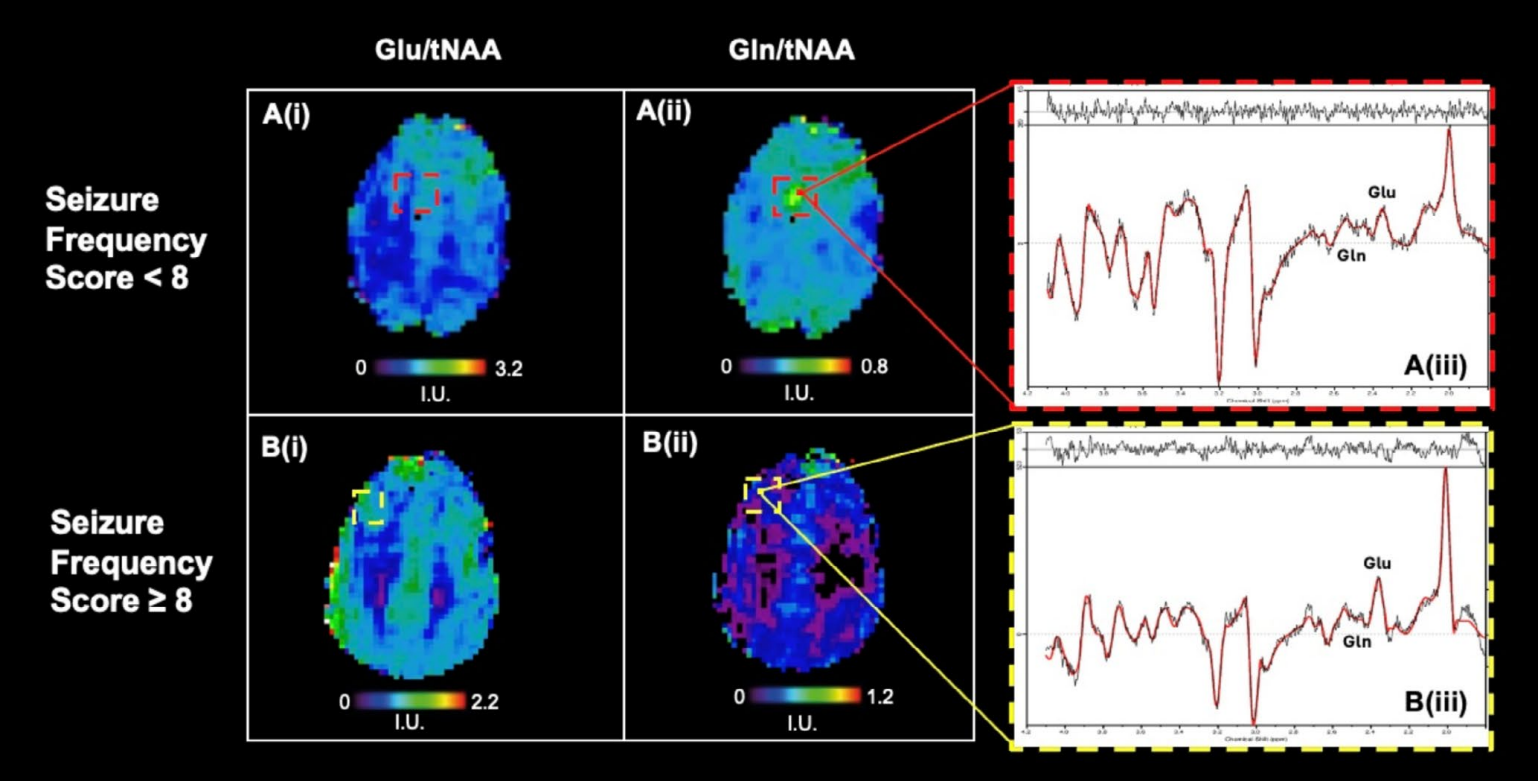
Glutamate and glutamine in relation to seizure frequency. Ratio maps of (i) Glu/tNAA, (ii) Gln/tNAA, and (iii) corresponding sample spectra in the suspected EZ. (A) Ratio maps of patient 23 with FCD in the left frontal lobe and SFS of 7 (i.e., monthly occurring seizures) showing a relative decrease of glutamate and increase of glutamine in the ROI. (B) Ratio maps of patient 18 with FCD 2a and SFS of 10 (i.e., daily occurring seizures). The sample spectra to the left showcase the subtle, although noticeable, relative increase of glutamate over glutamine when compared to A (iii). Glu, glutamate; Gln, glutamine; NAA, *N*‐acetyl‐aspartate + *N*‐acetyl‐aspartyl glutamate.

We next assessed the ratios of Glu/tNAA and Gln/tNAA of both groups in the epileptogenic zone compared to the contralateral hemisphere in relation to the seizure frequency; exemplary spectra are provided in Figure 6.

Glutamine increases were seen in 10/14 (71.4%) of PWFE with SFS ≤ 8 (i.e., weekly occurring seizures and less), whereas glutamate increases were observed only in 5/14 (35.7%). Conversely, patients suffering from a high seizure burden more frequently showed glutamate increases in 5/6 (83.3%), in contrast to glutamine in 1/6 (16.67%). However, these findings did not reach the level of significance in the Fisher's Exact Test (*p* = 0.057) for low seizure frequencies, *p* = 0.08 for high seizure frequencies. The mean age for PWFE with SFS ≤ 8 was 25.7 years (SD = 7.4) and 23 years (SD = 6.5) for PWFE > 8, which did not differ significantly between both groups (*p* = 0.607). Similarly, no significant differences could be uncovered in mean drug load between both groups (2.6 for SFS ≤ 8 vs. 2.3, *p* = 0.058).

**FIGURE 7:**
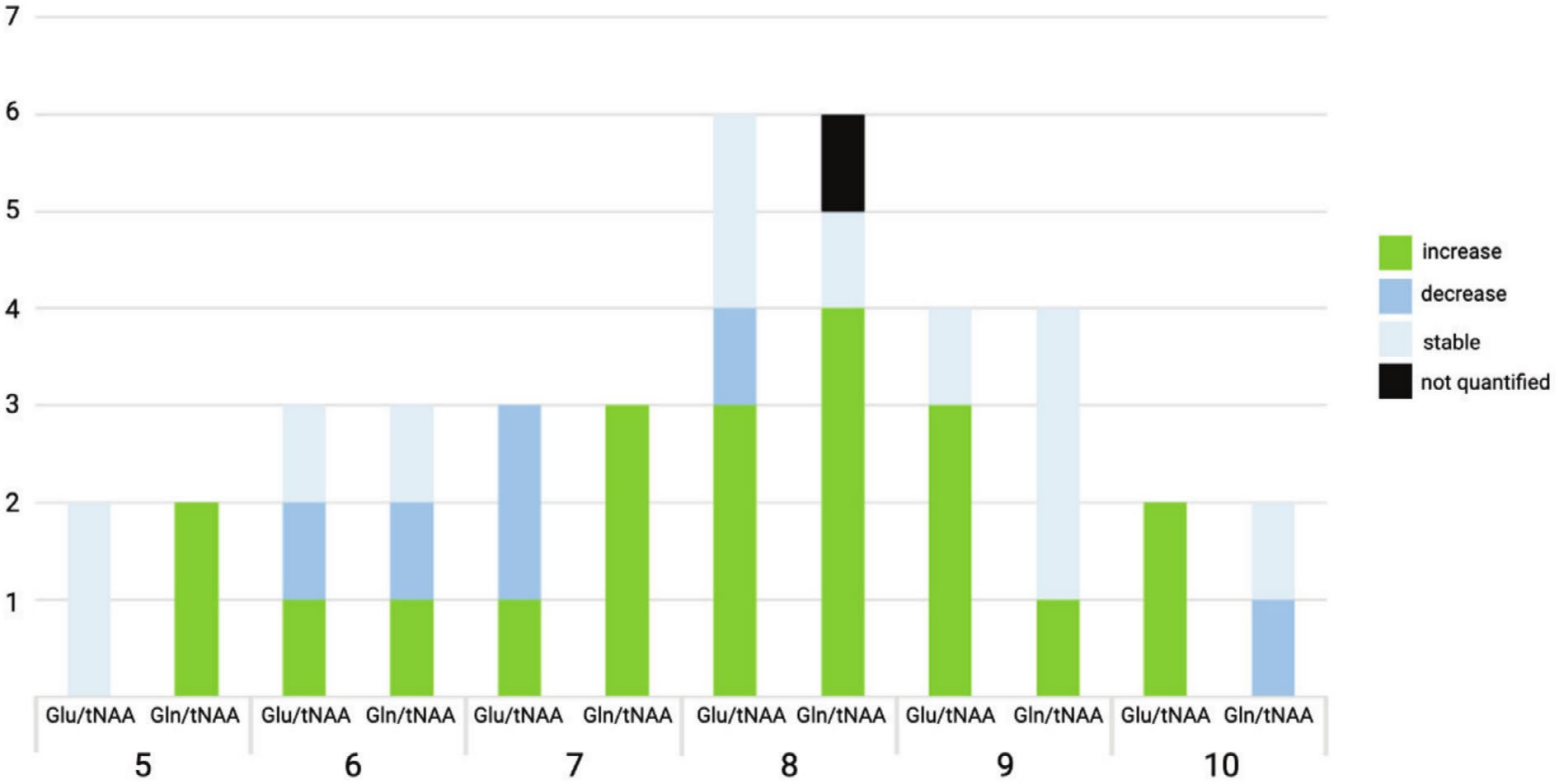
Glu and Gln in relation to the seizure frequency. X‐axis: SFS ranging from 5 (yearly occurring seizures) to 10 (daily occurring seizures). Y‐axis: Number of patients per group. Preliminary visual inspection of the maps indicated a tendency for Glu increases with higher seizure frequencies (i.e., daily occurring seizures), whereas a tendency to the inverse, namely, Gln increases, was observed in PWFE with lower seizure frequencies (i.e., monthly occurring seizures). Glu, glutamate; Gln, glutamine; SFS, Seizure Frequency Score.

These alterations are depicted in Figure 7 and key findings are summarized in Table 3. An extensive table providing the observed changes is provided in Table S4.

**TABLE 3:** Summary of key findings.

Metabolite Ratios			
	Increase	Decrease	Stable
Glu/tNAA	38.46%	23.08%	38.46%
Gln/tNAA	69.23%	15.38%	7.69%
tCh/tNAA	92.31%	0.00%	7.69%
mIns/tNAA	92.31%	0.00%	7.69%
tNAA/tCr	61.54%	0.00%	38.46%

Relation to Seizure Frequency				
SFS ≤ 8	Increase	Decrease	Stable	*p*‐value
Glu/tNAA	35.71%	28.57%	35.71%	0.057
Gln/tNAA	71.43%	7.14%	4.17%	

SFS > 8	Increase	Decrease	Stable	*p*‐value
Glu/tNAA	83.33%	0.00%	16.67%	0.080
Gln/tNAA	16.67%	16.67%	66.67%	

	Mean age (SD)	Mean drug load
SFS ≤ 8	25.7 (7.4) years	2.3
SFS > 8	23 (6.4) years	2.6
*p*‐value	0.607	0.058

*Note:* Top Row: Metabolic alterations in PWFE with visibly detectable lesions excluding MRSI‐negative cases. Bottom Row: Glu and Gln ratios in relation to the seizure frequency over the whole cohort. SFS > 8 signifies daily occurring seizures, whereas ≤ 8 corresponds to less than weekly occurring seizures. One missing value for Gln in SFS ≤ 8. *p*‐values derived using Fisher's Exact Test for relation to seizure frequency. Group differences in age and drug load were derived using a paired t‐test.

Abbreviations: Gln, glutamine; Glu, glutamate; mIns, myo‐inositol; Cr, creatine; SFS, Seizure Frequency Score; tCho, total choline; tNAA, *N*‐acetyl‐aspartate + *N*‐acetyl‐aspartyl glutamate.

## 4 Discussion

To the best of our knowledge, this is the first application of whole‐brain ultra‐high field MRSI at 7 T in focal epilepsy. Although our preliminary qualitative analysis suggests that profiling of metabolic changes across pathologies remains challenging, 7 T CRT‐FID‐MRSI shows promise in identifying metabolic alterations. Ratios normalized to NAA yielded an overall detection rate of 86.67% (13/15) in the EZ, whereas this was reduced to 80% (12/15) when normalized to tCr. This highlights that tNAA decrease is the main driver of the detection rate of metabolic alterations in our cohort. Despite NAA being less robust than creatine and more susceptible to pathologies, we chose to employ NAA normalization given its advantages for partially accounting for underlying brain atrophy and enhancing the sensitivity of lesion detection despite its limitations. Furthermore, a by‐product common to any increase in sensitivity of lesion detection is false positive findings, as seen in our data. This highlights the importance of multimodal clinical data to validate MRSI findings, which should be interpreted with caution in isolation and is supported by previous findings by our group demonstrating that concentration estimates of metabolites vary between healthy individuals, with mean coefficients of variation ranging from 9% to 11% [34]. The use of the contralateral hemisphere as a reference was applied to mitigate this effect.

In addition to assessing the utility of MRSI in detecting metabolic alterations in focal epilepsy, this work provides some insight into these over pathologies and clinical parameters. We observed that the directionality of change in assessed metabolites is at least partially dependent on clinical parameters such as seizure frequency, most notably in glutamate and glutamine. Although previous work has also been able to demonstrate this for Cr, visual assessment of the maps was insufficient to uncover such changes and will therefore require quantitative analysis. Conversely, metabolites that showed unidirectional changes across pathologies included mIns, tCho, and NAA. The discrepancy in influential factors of metabolic alterations is reflective of the nature of the assessed metabolites. Cr, Glu, and Gln are inherently linked to energy and neurotransmitter metabolism [35, 36], whereas mIns and tCho are considered markers of structural remodeling. Contrasting this principle, NAA, although indirectly linked to energy metabolism, consistently shows a reduction in epilepsy, particularly in FDG‐PET hypometabolic areas [17]. This may be indicative of a common denominator across some focal epilepsies, namely mitochondrial dysfunction. Impaired oxidative glucose metabolism is a frequent observation in many models of acquired epilepsy [37]. Decreases in interictal glucose utilization have been shown to be accompanied by a reduction in aspartate [33], the precursor to NAA, presumably reflecting impaired activity of the malate–aspartate shuttle [38]. However, neurons have a high degree of metabolic flexibility and inhibition of one metabolic pathway leads to compensatory activity of other pathways [35]. In an in vitro model of epileptogenesis, interictal neuronal activity known as paroxysmal depolarization shifts (PDS) [39], has been shown to lead to a decrease in neuronal glucose metabolism and activity of malate–aspartate shuttle. It also leads to a compensatory increase in glutaminolysis, a process of glutamine/glutamate breakdown in the tricarboxylic acid (cycle [38]). Furthermore, it has been shown that the glutamine used for neuronal glutaminolysis is shuttled to neurons by glial cells [38], which maintain high levels due to their ability to synthesize it de novo [40]. Therefore, it can be argued that in an interictal state, neurons hypometabolize glucose, leading to reduced levels of NAA and Glu. The observed increase in interictal Gln may reflect increased synthesis in glia, which may enable the compensatory increase in neuronal glutaminolysis to maintain sufficient energy production. Notably, MRSI does not distinguish between intra‐ and extracellular metabolite concentrations. However, previous work using complementary research techniques such as microdialysis provides approximations for extracellular glutamate concentrations, which support the interpretation of MRS signals as primarily reflecting intracellular concentrations [41].

This metabolic background is supported by plasma concentrations of Glu and Gln in PWE. While increased Glu plasma levels were seen in uncontrolled epilepsy, elevated levels of plasma Gln were observed when seizures were controlled [42]. Although all PWFE included in our study were drug‐resistant, seizure frequencies span from yearly to daily occurring seizures, allowing for a comparison within the cohort. Analogous to the findings above, we saw increases of Glu in PWFE with high seizure frequencies and Gln increases in low seizure frequencies in our preliminary analysis. Though these changes did not reach the level of statistical significance in our limited cohort, the *p*‐values narrowly exceed this marker and may indicate a trend and warrant further quantitative analysis. Unfortunately, the timepoint of the last seizure prior to measurement, necessary to validate this hypothesis, was not available in this study. Further underpinning the complexity of energy metabolism in epilepsy, other groups have observed slowing of the glutamate‐glutamine cycle in hippocampi in mesial temporal epilepsy [23], potentially contributing to delayed clearance of glutamate and higher glutamate concentrations following seizures, and increased glutamate connectivity as demonstrated in a network analysis at 7T [43]. Nonetheless, characterization of glutamate and glutamine levels in epilepsy has thus far delivered divergent results as presented in a recent review [24] highlighting the necessity of interpreting these data in light of clinical measures.

## 5 Limitations

Due to the limited cohort size, intergroup comparisons across pathologies were not possible. Moreover, the lack of knowledge of the last seizure prior to measurement impedes our ability to interpret the observed changes. Furthermore, the effect of antiseizure medication was not considered in this analysis. However, as all PWFE included in this study were drug‐resistant, most have had multiple therapeutic schemes, thereby potentially spreading the effect size across the cohort. Future MRSI research, particularly of mono‐therapeutic schemes, may lend insight into the metabolic effects of ASM. Last, the lack of comparable studies in this field limits the validity of our findings, and future research may aid in contextualizing our results.

Methodologically, our analysis is limited by its qualitative nature. Future work will focus on quantitative assessment of average metabolic alterations in segmented EZ and perilesional zones, in which visual inspection is insufficient. Furthermore, B_0_‐inhomogeneities, inherent to the ultra‐high‐field MRSI research, severely impeded the assessment of basal locations, such as mesial temporal lobes, essential in epilepsy research, necessitating their exclusion from further analysis. Though the use of parallel transmission coils (pTx) has markedly improved B_1_‐field inhomogeneities in structural imagin*g* [44], B_0_ shimming is paramount in CRT‐FID MRSI for improved MRSI signal and the focus of ongoing work at our center.

## 6 Conclusion

In this study, we successfully generated high‐resolution, whole‐brain maps in epilepsy using 7 T CRT‐FID MRSI showing high detection rates of metabolic alterations across multiple pathologies corresponding to the EZ and lend some insight into metabolic alterations in relation to clinical parameters. Although we were not able to demonstrate group differences across etiologies, our findings suggest that, while metabolites such as mIns and tCho show unidirectionality of changes, Glu and Gln show dependency on the seizure frequency. Furthermore, we propose that Glu and Gln share an inverse relationship in the EZ dependent on the seizure interval, highlighting the necessity of spectral separation of these metabolites. With the availability of new MRSI methods at 7 T, in vivo validation of this hypothesis is possible for the first time and further work in this area by multiple groups may greatly contribute to understanding disease mechanisms. Conclusively, our preliminary analysis suggests that 7 T CRT‐FID MRSI shows promise not only as a feasible method for detecting metabolic alterations, but also may aid in understanding underlying complex metabolic pathomechanisms in epilepsy.

## Conflicts of Interest

The authors declare no conflicts of interest.

## Supporting information


**Table S1:** Clinical data of included patients. All patients underwent prolonged video‐EEG‐monitoring at a tertiary epilepsy center for the assessment of the suspected EZ. This also included the assessment of the seizure semiology, clinical 3 T MRI with a dedicated epilepsy protocol, neuropsychological testing and FDG‐PET. The seizure frequency was estimated using the Seizure Frequency Score [27]. MRI‐negative excluded incidental findings unrelated to the suspected EZ. Where available, histopathological verification of the suspected pathology is supplied. In cases without histopathological assessment, the radiological diagnosis was used as a classifier. Drug load relates to the number of antiseizure medication prescribed at the time of the MRI.
**Table S2:** Metabolite trends in NAA ratios in lesional epilepsy.
**Table S3:** Metabolite trends in NAA ratios in MRI‐negative PWFE.
**Table S4:** Metabolite trends in NAA ratios with increasing seizure frequency.
**Table S4:** Overview of Minimum Reporting Standards for in vivo Microscopy used in this study [29].

## Data Availability

The data that support the findings of this study are available on request from the corresponding author. The data are not publicly available due to privacy or ethical restrictions.
